# New horizons in systems engineering and thinking to improve health and social care for older people

**DOI:** 10.1093/ageing/afae238

**Published:** 2024-10-29

**Authors:** Navneet Aujla, Tricia Tooman, Stella Arakelyan, Tim Kerby, Louise Hartley, Amy O’Donnell, Bruce Guthrie, Ian Underwood, Julie A Jacko, Atul Anand

**Affiliations:** Population Health Sciences Institute, Faculty of Medical Sciences, Newcastle University, Newcastle upon Tyne, UK; School of Psychology and Vision Sciences, College of Life Sciences, University of Leicester, Leicester, UK; Advanced Care Research Centre, Usher Institute, University of Edinburgh, Edinburgh, UK; Advanced Care Research Centre, Usher Institute, University of Edinburgh, Edinburgh, UK; Edinburgh Systems Ltd, Edinburgh, UK; The Usher Institute, University of Edinburgh, Edinburgh, UK; Edinburgh Systems Ltd, Edinburgh, UK; Population Health Sciences Institute, Faculty of Medical Sciences, Newcastle University, Newcastle upon Tyne, UK; Edinburgh Systems Ltd, Edinburgh, UK; Advanced Care Research Centre, Usher Institute, University of Edinburgh, Edinburgh, UK; School of Engineering, University of Edinburgh, Edinburgh, UK; The Centre for Medical Informatics, The Usher Institute, University of Edinburgh, Edinburgh, UK; Advanced Care Research Centre, Usher Institute, University of Edinburgh, Edinburgh, UK; BHF Centre for Cardiovascular Science, University of Edinburgh, Edinburgh, UK

**Keywords:** systems engineering, systems thinking, multimorbidity, older people

## Abstract

Existing models for the safe, timely and effective delivery of health and social care are challenged by an ageing population. Services and care pathways are often optimised for single-disease management, while many older people are presenting with multiple long-term conditions and frailty. Systems engineering describes a holistic, interdisciplinary approach to change that is focused on people, system understanding, design and risk management. These principles are the basis of many established quality improvement (QI) tools in health and social care, but implementation has often been limited to single services or condition areas. Newer engineering techniques may help reshape more complex systems. Systems thinking is an essential component of this mindset to understand the underlying relationships and characteristics of a working system. It promotes the use of tools that map, measure and interrogate the dynamics of complex systems. In this New Horizons piece, we describe the evolution of systems approaches while noting the challenges of small-scale QI efforts that fail to address whole-system problems. The opportunities for novel soft-systems approaches are described, along with a recent update to the Systems Engineering Initiative for Patient Safety model, which includes human-centred design. Systems modelling and simulation techniques harness routine data to understand the functioning of complex health and social care systems. These tools could support better-informed system change by allowing comparison of simulated approaches before implementation, but better effectiveness evidence is required. Modern systems engineering and systems thinking techniques have potential to inform the redesign of services appropriate for the complex needs of older people.

## Key Points

- Systems engineering and thinking seek improvement through a focus on people, system understanding, design and risk management.
- A whole-system approach may overcome some of the limitations observed with small-scale quality improvement.
- Newer techniques include human-centred design, soft-systems methods, and using routine data to model and simulate systems.

## Introduction

Health and social care systems have not fully adjusted to a fundamental shift from caring for people with single-organ pathology to supporting the complex needs of an ageing population with multiple interacting conditions. Most older people are now living with multiple long-term conditions (MLTC), but evidence-based healthcare guidelines for multimorbidity have only emerged in the last decade [1] and innovation is often wedded to the paradigm of single-condition care pathways [2]. MLTC are more common in the less affluent, and poorly designed systems of care may further exacerbate inequalities [3, 4]. Social care remains undervalued [5, 6] and promised systems integration with health services is far from universal despite its prioritisation at a policy level [7]. Service pressures continue to rise with persistent markers of perceived ‘system failure’ for older people, such as an inability to access services when needed, ‘delayed discharges’ from hospital and more frequent, early readmissions [8].

There is a clear challenge in adapting systems of care to manage the additional complexity of frailty and MLTC in older people. Creative solutions are needed across both health and social care at a time when funding is likely to remain constrained. Marginal efficiency improvements in existing services—doing more of the same but ‘better’—are harder to find, and many advocate for wider system redesign. In 2017, the Royal Academy of Engineering published ‘Engineering Better Care’ in partnership with the Royal College of Physicians and the Academy of Medical Sciences [9]. This highlighted opportunities to improve services through an engineering approach. Specifically, the report observed that existing services were often not person-centred, that changes that were frequently implemented before demonstrating effectiveness, that risk management was often limited and reactive, and that design was rarely creative. Systems engineering and systems thinking approaches from a range of sectors like manufacturing and transportation have influenced healthcare improvement work over the last three decades. In this paper, we will describe new horizons in the use of systems engineering within the delivery of health and social care for older people. We emphasise how its focus on the meticulous integration of people, systems, design and risk management differs from existing improvement methods and examine future opportunities.

## Quality improvement

Continuous improvement methodologies are already mainstream within healthcare delivery and will be familiar to most clinicians through audit and quality improvement (QI). These varied approaches share common structures to implement change and measure its effect. The Health Foundation describes QI as the ‘systematic and coordinated approach to solving a problem using specific methods and tools with the aim of bringing about a measurable improvement’ [10]. These ideas were first introduced in the 1990s by the Institute for Healthcare Improvement (IHI) in their ‘Model for Improvement’ and this concept has spread from the US across the world. However, early QI projects appeared remote from clinicians’ understanding of patient needs, were often narrow in focus, took far too long to progress, and were run by individuals without skills to motivate frontline practitioners [11].

In response, the IHI proposed QI collaboratives and these have now become a mainstay of improvement organisations, demonstrating effectiveness in diverse healthcare settings from the US to Africa [12, 13]. These involve structured, collaborative working with shared learning and experience across individuals and groups from different organisations, often supported by experts or ‘coaches’ [14]. However, a recent evaluation of the UK’s Acute Frailty Network QI collaborative to improve access to Comprehensive Geriatric Assessment (CGA) for older patients with frailty across 66 hospitals demonstrated no effect on length of stay, mortality, institutionalisation or readmission risk [15]. This is despite research evidence to support the effectiveness of CGA in this population [16]. Here, the implementation strategy for delivering change across multiple hospitals’ systems was considered inadequate [15, 17]. Setting up a collaboration will not overcome challenges of capacity or deficiencies in the use of core systems techniques like managing behaviour change and organisational psychology [14].

The principles of QI include a wide systems perspective [18, 19], but delivery often occurs in isolated small process improvement projects despite these processes existing within increasingly fragile systems that are burdened by systemic issues. For example, a whole-system view would attempt to understand and mitigate the impact of social determinants of health (e.g. poverty) on access to centralised hospital-based services, but this might be considered out of scope in a project focused on redesigning a single-disease care pathway [20]. As more people live with MLTC, health and social care workforces require training and education to support increased patient complexity as a necessary foundation for any system change. To enhance the experience of healthcare for people with MLTC, communication and delivery barriers between siloed specialities must be challenged to deliver whole-system improvement.

These challenges to improving healthcare delivery have been widely recognised. The World Health Organisation (WHO) Global Learning Laboratory for Quality shares knowledge to help overcome common barriers to delivering healthcare improvement [21]. In the UK, NHS England’s recent Delivery and Continuous Improvement Review recommended better alignment of improvement work [22]. This has resulted in the creation of NHS IMPACT (Improving Patient Care Together) as a new hub focused on developing a single, shared NHS approach backed by a national improvement board [23].

## What are systems engineering and systems thinking?

The International Council on Systems Engineering defines their field as an interdisciplinary academic approach to ‘enable the successful realisation, use, and retirement of engineered systems, using systems principles and concepts and scientific, technological and management methods’ [24]. A ‘system’ describes the arrangement of multiple interacting components that together exhibit behaviour that goes beyond the actions of individual constituents—where the sum is greater than its parts. Health and social care are generally considered complex adaptive systems in that they comprise collections of individual actors, organisations and sectors that are nested within wider social, political and economic contexts and sub-systems and whose actions are both interconnected and often unpredictable or nonlinear [25–29].

A systematic review and meta-analysis of systems approaches to health service design, delivery and improvement identified 35 studies, but variation in settings and implementation techniques limited recommendations for optimum approaches [30]. Notably, only one study specifically focused on older adult care (optimising a hip fracture pathway [31]), although many addressed issues like patient flow, emergency care and prescribing safety, which impact all specialities. The authors reported an overall benefit in service and resource use outcomes following system-level interventions but acknowledged a significant risk of bias in before-and-after analyses where the Hawthorne effect might have influenced findings. This is where the act of being observed changes the behaviour of healthcare staff in a way that might not be sustained after the study is completed. There were also only two randomised controlled trials of a systems approach identified, focused on reducing HIV transmission in Africa [32] and promoting smoking cessation in the USA [33]; both studies, however, did demonstrate benefits against control groups.

There is no single method of delivering a systems approach and many models have been developed over the past 100 years since the concept was first described in manufacturing industries. Techniques have evolved to include broader stakeholder engagement, system modelling and simulation [34]. Some of the most important systems engineering methods that have been used successfully in healthcare include project management models, process flow analyses and discrete event simulation [35]. Systems engineering was a response to the need to consistently manufacture increasingly complex products. Although some ‘hard’ engineering processes were successful when applied to systems like healthcare IT infrastructure, it was quickly appreciated that a ‘soft’ systems methodology was required for managing the complexity of healthcare processes for patients with very differing needs [36]. A core systems engineering concept is the breaking down of complexity to map a process from the smallest possible replicable unit—often a patient-practitioner interaction. These microsystems build into mesosystems (e.g. GP practices, wards or care homes), which are influenced by macrosystems (e.g. hospital management, government policy). This ‘whole system’ view is termed a ‘work system’ by engineers to represent the operational interaction between people, equipment and the environment.

**Figure 1:**
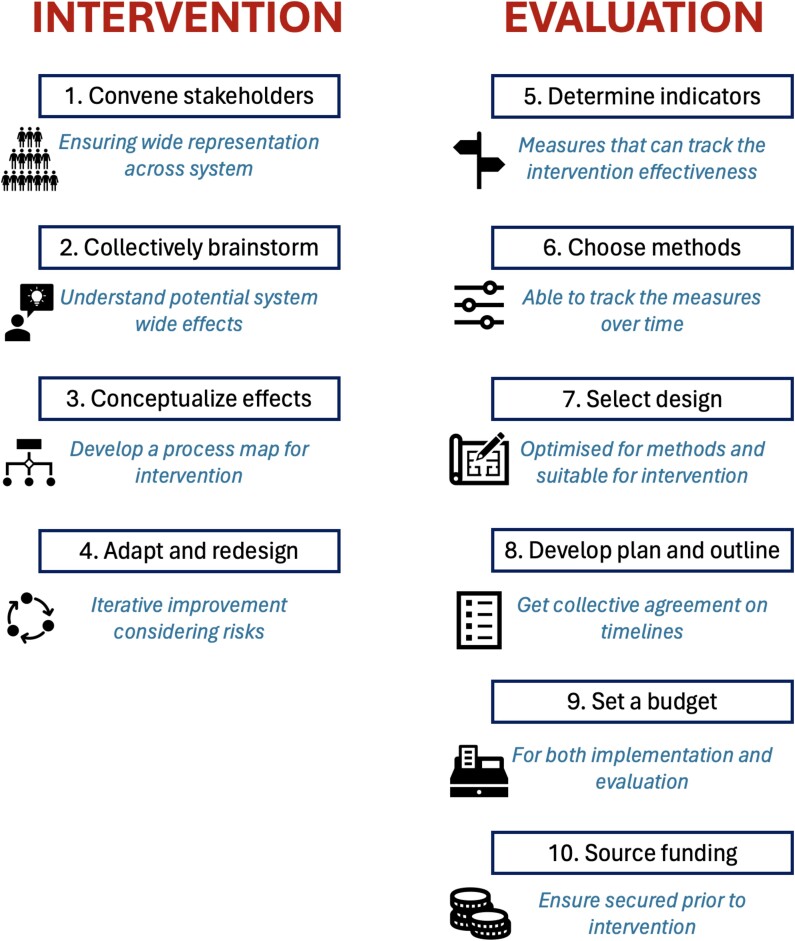
WHO ‘Ten Steps to Systems Thinking’ covering both intervention and evaluation design. Figure adapted from the WHO report on Systems Thinking for Health System Strengthening [88].

Common to these approaches is ‘systems thinking’—a focus on understanding the underlying relationships and characteristics of work system components, most notably the people central to its function. Systems thinking promotes tools that carefully map, measure, and interrogate the dynamics of complex systems. The WHO has recognised the importance of these approaches to deliver its global goals, producing a 10-step framework for introducing systems thinking to strengthen health systems (see Figure 1) [37]. Most of these steps feature in traditional QI approaches, but the framework attempts to strengthen upfront consideration of whole-system effects of interventions, and robust evaluation with clearly measurable outcomes. Systems engineering and systems thinking are still evolving in healthcare and have rarely been implemented within social care settings. As early as 2005, The US National Academy of Engineering and the US Institute of Medicine published a joint report recommending the systematic application of systems engineering approaches for reforming the US healthcare delivery system [38], but transformative improvements remain an unrealised goal. Reimbursement practices and regulations in the US have been cited as disincentives for investing in quality and systems improvement, but such approaches might be the only way to address unsustainable acceleration in the costs of care [35].

Table 1 summarises the numerous tools that have been adopted under a healthcare improvement umbrella that broadly include systems approaches with case examples. These methods share a continuous improvement philosophy with iterative development of solutions to improve quality of care. However, most were not created for a health or social care context, so an apparent focus on maximising financial returns or customer value can feel uncomfortable for many practitioners.

**Table 1:** Summary of QI tools that include a systems approach.

Tool	Origins	Key features	Use cases examples in healthcare
Total Quality Management	Conceived in America; initially implemented in Japanese manufacturing	Focus on team responsibility rather than driving improvement from top-down management [67]. This promotes a strong focus on education, communication and training in the workforce.	In a training programme responding to medication errors across 24 US hospitals, a Total Quality Management approach was used to address safety reporting and activity (e.g. team huddles and structured debriefs). Questionnaire responses demonstrated improved safety culture and team behaviour measures across >3000 staff who responded when compared to responses from staff at 13 control hospitals [68].
Lean	Toyota’s car production system in Japan [69].	Continuous improvement of work processes and flow by reducing or removing low-value activities that lead to waste, delay, or errors.	Used in many healthcare settings, particularly in emergency care patient flow and efficiency [40, 70]. For example, White *et al.* demonstrated cost-neutral improvements in a single Emergency Department’s performance using Lean approaches to reorganise use of space and flow patterns for patients after arrival to reduce wasteful waiting. Median length of stay in the Emergency Department reduced by 15 minutes and use of examination rooms by 34 minutes per patient, while these measures were unchanged in a control department over the same period [71].
Kaizen	Conceived in Japan, literally meaning ‘good change.’	Focus on culture change and flattened management hierarchy [72, 73]. Workers at all levels of service should feel empowered to challenge the status quo and see opportunities to improve processes whenever hearing the phrase ‘that is just how it’s done’.	An oncology clinic model was developed to reduce waits for follow-up CT scanning, which were overdue against targets for 28% of patients at baseline. A root-cause analysis fed into a Kaizen group of key clinicians and administrators to develop and roll out a new workflow that virtually eliminated overdue scans (<1%) within 10 weeks. The analysis period was extended to 10 months after implementation when overdue scans had risen slightly but still represented a sustained 75% reduction from baseline levels [74]. Qualitative analysis of Kaizen cycles for multiple improvement projects across 26 Finnish hospitals suggested sustained improvement in the majority of units following intense 5-day planning and design events with follow-up of implemented change activity over the next 1–3 months [75].
PDSA	Industrial PDCA (PDSA) approach [76, 77].	Four iterative stages of learning and improvement: the ‘Plan’ stage identifies what change is required, which is then tested in the ‘Do’ stage, the success of which is examined in the ‘Study’ stage, and the ‘Act’ stage identifies areas for improvement or adaptation to inform the next cycle [78].	Widely used in QI programmes, particularly in the UK and America. The PDSA approach is a core part of the IHI Model for Improvement [79]. For example, the PDSA approach has been used to improve bone health guideline adoption for patients referred to a movement disorder clinic [80] and to improve care home staff confidence in using an early warning tool for deterioration in residents [81]. In these cases, each cycle of improvement responded and adapted the intervention according to barriers and emergent issues identified in the initial roll-out of new processes. For example, staff in the care home project were unable to consistently attend training sessions and so a reminder prompt and named invitation lists were added to the process.
Six sigma	Motorola semiconductor manufacturing processes.	Continuous improvement methods focused on reducing defects to a level of quality close to perfect, or specifically fewer than 3.4 defects per million processes [82, 83]. It follows a stepwise approach to define, measure, analyse, improve and control.	An Irish hospital used this approach to improve delirium screening in older adults using the 4AT tool in an Emergency Department setting. The process defined prioritised interventions including education, revised documentation and team huddles. Delirium screening rates on sample audits improved from 16% to over 90% with sustained improvement several weeks after the intervention completed [84].
Statistical process control	Industrial manufacturing	Defining a true signal of change against a background of potential noisy natural variation [85]. This uses control charts, run charts and flow diagrams to support the implementation of other QI frameworks [86].	Using data visualisation through techniques such as statistical process control has been a key component of NHS England’s ‘Making Data Count’ campaign [87].

## The challenge of systems change

Implementation of relatively simple, well-defined interventions such as the WHO Surgical Safety Checklist are perhaps best amenable to QI approaches and this can have dramatically positive effects [39]. However, the success of these projects has led to assumptions that a checklist approach can be applied almost irrespective of the kind of complexity and heterogeneity that characterises care for many older people. Approaches like Lean are likely to remain best suited to well-defined, simple technical pathways [40] and have inconsistent effects when applied to more complex areas [41]. It could be argued that a relentless focus on waste and inefficiency does not necessarily improve quality or delivery of person-centred care, and many healthcare applications have acknowledged the need to enhance these elements within Lean working.

The number and complexity of stakeholder relationships involved in the delivery of health and care to an older person with complex health or social care needs may be orders of magnitude greater than in the optimisation of a manufacturing process of a physical product [10]. Reducing variation of inputs by standardising materials makes sense in an industrial process, but if applied to health and social care, it would promote inequitable disregard of ‘complex’ people with MLTC. Most improvement techniques have, therefore, placed greater importance on a comprehensive stakeholder engagement phase when adapting to healthcare, but there remains evidence of a rush to implement change [9]. In an independent evaluation of the Safer Clinical Systems QI programme, The Health Foundation reported ‘many system defects that were not tractable to improvement using QI methods based on Plan-Do-Study-Act (PDSA) cycles led by small clinical teams’. The challenges were simply too systemic and structural to be solved by a narrow process or small-scale system improvement. Many outcomes felt to be important for evaluation also turned out to be immeasurable, adding uncertainty to the evaluation [42]. Critics argue that the PDSA approach encourages such a narrow focus on local implementation of change, with the potential for causing unintended emergent consequences in the wider system [43].

Thus, despite strong advocacy for QI methodology, the hard evidence to support its effectiveness is, at best, mixed. In part, this may reflect poor adherence to gold standard QI processes, but it also suggests challenges that impede implementing these approaches within healthcare services [44]. A culture of QI may encourage multiple small-scale projects that seek to address downstream effects because whole-system reform is seen as impenetrable. This has been described as the ‘problem of many hands’, and indeed, it has been argued that multiple local QI projects may paradoxically worsen outcomes, including by unintended worsening of patient safety [45]. Without embedded systems change, short-term improvement may be achieved by motivated individuals but be difficult to sustain without continued focus. This critical challenge for all improvement work is well recognised [46]. Dixon-Woods and Martin have argued for the need to change healthcare QI in four ways: to embrace system-level change, to stop looking for ‘magic bullets’, to build upfront capacity to allow scale and spread; and to think about long-term programmes of activity rather than individual projects [44].

## Newer systems engineering approaches for healthcare

**Figure 2:**
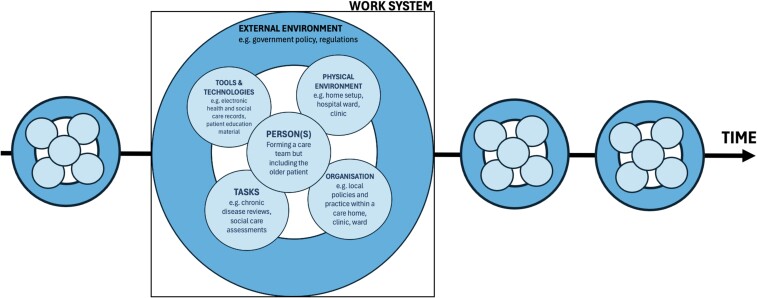
SEIPS 3.0 model. The work system comprises six components. The updated model introduces the concept of spatio-temporal distribution, with a person experiencing multiple work systems over time due to multiple interactions with different services, which is particularly common in the management of MLTC. Adapted from Carayon *et al*. [47]

The Systems Engineering Initiative for Patient Safety (SEIPS) model was first described in 2006, but a recently described version 3.0 includes new human-centred design elements [47]. There are six core elements to the SEIPS model (see Figure 2) that are intended to create a full understanding of a healthcare system from individuals up to government policy. Examples of healthcare applications of SEIPS include improving medicines management on hospital discharge [48] and optimising hand hygiene in wards [49]. In an analysis by Werner *et al.*[50], the SEIPS approach identified process issues in medication management for older people discharged from hospitals to care homes. Through an interview study, the authors identified poorly defined roles and responsibilities within teams and a need to extend medication oversight for these complex patients beyond the day of hospital discharge. Prior task-orientated improvement work had not identified these team dynamic issues [48]. As SEIPS was implemented in more settings, it became clear that its view of a ‘work system’ unit was too simplistic for many people with MLTC, who frequently move between care providers. The SEIPS 3.0 framework added this appreciation of a series of interacting work systems for a person being distributed over time and space. The authors also recognised that identifying complexity is only the start of a process, and this does not necessarily lead to solutions. Bringing together multiple stakeholders from disparate organisations may introduce logistic barriers and getting meaningful outputs from diverse groups might require new approaches.

One response to these issues is Soft Systems Methodology (SSM), which is an organised but more flexible approach to tackling complex problems [51]. SSM uses interconnected stakeholder engagement to define ‘models of action’ as the basis for solution co-design. Although there is a risk that consensus is never reached between disparate groups, sharing ownership of solution design can enhance group learning and understanding of each other’s challenges. For example, Emes *et al.* [52] looked at hospital discharge processes and identified the frequent tension between a ‘care model’ (driven by clinicians’ views of readiness for discharge) and a ‘flow model’ (led by managers focused on hospital capacity). Using SSM, consensus was achieved on three feasible interventions that were perceived to have shared value to proponents of both models: introducing a single point of recording social care needs on admission, reducing the burden of health needs assessment forms, and introducing a dedicated daily huddle of relevant decision-makers to progress discharges for more complex patients. Implementation achieved a 41% reduction in the time waiting for discharge when medically fit [53].

**Figure 3:**
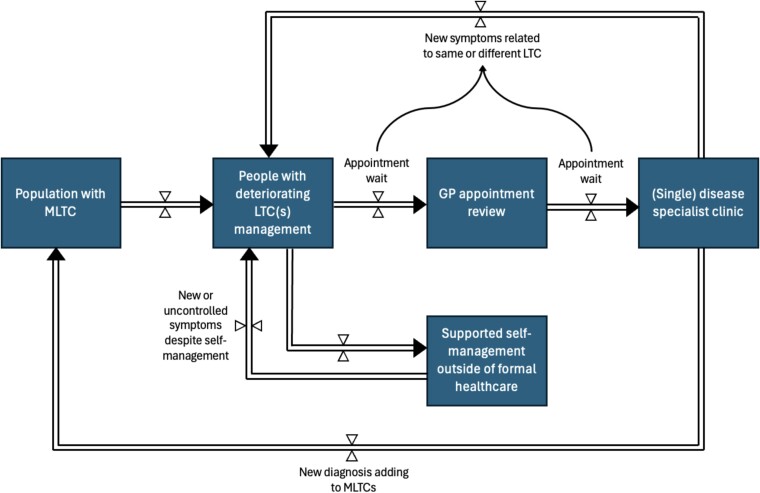
Simplified stock and flow diagram to show patients with deteriorating control of MLTC. Patients flow (arrows) between stocks (blocks). In this example, wait times for assessment are potential periods for further symptom development which may precipitate a cycle of deterioration.

Increasing access to routine electronic health data is shaping newer systems engineering approaches, particularly in the fields of systems modelling and simulation. In a world of digital records, many, most or occasionally, all processes within a person’s health and care journey are now captured with a timestamp, such as prescriptions, referrals, appointments, care visits and movements between hospital wards. This allows the modelling of whole system dynamics using mathematical approaches to simulation that may highlight areas of particular weakness or bottlenecks [54]. Stock and flow diagrams can help to visualise these models, where the ‘stock’ represents the store of a system component (e.g. a group of people with a particular health condition) and the ‘flow’ is the movement between different stocks over time (see Figure 3). In this example, a simplistic view of a GP referral of a patient to a specialist disease clinic is expanded, to understand activity during waiting periods for the patient, including interactions with supported self-management. Expanding these diagrams with real data of the size of each stock (e.g. numbers waiting specialist review) and rate of each flow (e.g. numbers accessing self-management) can help to identify the most important rate-limiting variables as targets for tests of change. In a social care setting, the flow may represent the transition from a low-level of care requirement stock to a higher state of dependency.

Alternative methods such as discrete-event simulation (DES) are helpful when the sequence of pathways is highly reproducible and sequential, such as specific activities undertaken from the moment a person arrives at an Emergency Department [55]. In one example from the US, DES was used to model optimum staffing shift patterns and a new model of earlier physician review after patient arrival [56]. A key advantage of simulation is the ability to explore the effect of proposed system change before implementation. Simulation software was used to model different patient attendance rates before committing to redesign staff rotas or reorganisation of physical space. The resulting improvement work was associated with a reduction in median length of ED stay by 23% despite patient volumes increasing by 22%. Other relevant examples include the understanding of delayed discharges in older patients [57] and simulating different staffing scenarios for social care delivery to older people in the community [58] and in continuing care units for dementia [59]. With advancing computing power and newer machine and deep learning methods, the power of these techniques to more accurately model variation is likely to improve further. However, even the most advanced techniques will not overcome deficiencies in data capture and accuracy, so it is imperative that electronic systems are designed to capture and label these key system transitions [60]. It also remains unclear if models can usefully capture the complexity and heterogeneity of less disease-specific care pathways, many of which affect older people and those with MLTC.

## Discussion

It has been 50 years since Rittel and Webber first described their concept of ‘wicked problems’—challenges without a clear aim or solution, which can appear intractable partly because all potential solutions can themselves create other wicked problems [61]. Many apply this label to describe the design of services appropriate for an ageing population with increasingly complex health and social care needs. Others see potential to reimagine the systems in which we operate. QI and continual improvement methods are essential to the development of services for older people, and many of these approaches have a basis in systems engineering philosophy. However, it might be argued that the sheer desire of so many people working in health and social care to improve and change their services has weakened whole systems thinking; the desire to address obvious and visible failures encourages improvement work that is separated from an appreciation of system impact. Each health or social care professional is ultimately best placed to understand and control their own sphere of practice, and it is a real challenge to add higher-level system thinking to service redesign. But without this wider lens, it is more difficult to truly understand the opportunity costs of an intervention– that is, the potential to achieve even better outcomes when applying available resources to an alternative strategy. Modern systems engineering methods of modelling and simulation provide a framework for appraising the likely impact of multiple options for change, accepting that this more considered approach is slower and requires good underlying data.

An important benefit of a systems engineering approach is a greater emphasis on active risk management [62]. Our health and care services are rightly influenced by a need to learn from harm, a framework ingrained by the seminal publication of ‘To Err is Human’ in 2000 [63]. While such postmortem review of significant events is important for critical failures, this mindset neglects the vast majority of practice that goes well without incident or harm [64]. The systems engineering approach to risk management seeks to learn from good practice and existing adaptability of systems to proactively manage risks when things occasionally go wrong. This brings risk management much earlier in the process of improvement and encourages a culture of ‘premortem’—identifying how a project could fail throughout its design while trying to mitigate these risks [43]. Uncertainty must be embraced in all systems change, particularly when trying to influence complex adaptive systems like healthcare. This is a key component of systems thinking. Optimism bias amongst leaders of change can risk assumptions of future benefit, ignoring the risks of unpredictable and potentially harmful emergent behaviours in response to system change. The best improvement projects will actively monitor for such harms and adapt quickly.

System change is rarely tested to the same research standard as other interventions, such as novel therapeutics, but may have similarly profound positive or negative effects on individuals. Such research has challenges. For example, it is often unsafe or impractical to randomise patients to new care pathways within the same institution or system. However, cluster-randomisation at the provider, rather than person, level is a valid alternative. Successful delivery of evidence-based care from carefully controlled trial environments to complex and sometimes chaotic health and social care systems is difficult. The field of implementation science recognises this gap between evidence and delivery [65]. Its focus is on the method of effectively delivering change rather than just the components of change, and it is, therefore, well aligned with systems engineering approaches. The end goal must always be an improvement in meaningful outcomes for people being cared for by the system, not simply successful implementation of a planned change as determined by process measures. A wider appreciation of socio-political contextual factors from the field of complexity science may also improve the chances of achieving successful system change [66].

Recognising the opportunities of systems engineering, the National Institute for Health and Care Research and the UK Research and Innovation Engineering and Physical Sciences Research Council launched an Innovation Hub programme in 2022, intending to fund multiple systems engineering centres across the UK specifically focused on developing services for people with MLTC. This is likely to build capacity in both systems engineering research methodology and implementation over the next decade.

## Conclusion

Systems approaches fundamentally require an understanding of the dependencies between components within a complex web of people and processes. A deeper integration of modern systems engineering and thinking could augment QI initiatives for designing health and social care services appropriate for the complex needs of older people, but better evaluation and evidence are needed.
