# Correction to: New horizons in systems engineering and thinking to improve health and social care for older people

**DOI:** 10.1093/ageing/afag222

**Published:** 2026-07-12

This is a correction to: Navneet Aujla, Tricia Tooman, Stella Arakelyan, Tim Kerby, Louise Hartley, Amy O’Donnell, Bruce Guthrie, Ian Underwood, Julie A Jacko, Atul Anand, New horizons in systems engineering and thinking to improve health and social care for older people, *Age and Ageing*, Volume 53, Issue 10, October 2024, afae238, https://doi.org/10.1093/ageing/afae238

In the originally published version of this manuscript, the fifth and seventh co-author’s affiliation information was incorrect.

Louise Hartley and Bruce Guthrie should be affiliated to institution 3, “Advanced Care Research Centre, Usher Institute, University of Edinburgh, Edinburgh, UK”, instead of institution 4, “Edinburgh Systems Ltd., Edinburgh, UK”.

These details have been corrected only in this correction notice to preserve the version of record.

